# Cutaneous Immunoprofiles of Three Spotted Fever Group Rickettsia Cases

**DOI:** 10.1128/IAI.00686-19

**Published:** 2020-03-23

**Authors:** Na Jia, Hong-Bo Liu, Yuan-Chun Zheng, Wen-Qiang Shi, Ran Wei, Yan-Li Chu, Nian-Zhi Ning, Bao-Gui Jiang, Rui-Ruo Jiang, Tao Li, Qiu-Bo Huo, Cai Bian, Xiong Liu, Yi Sun, Lian-Feng Li, Qian Wang, Wei Wei, Ya-Wei Wang, Frans Jongejan, Jia-Fu Jiang, Ju-Liang Song, Hui Wang, Wu-Chun Cao

**Affiliations:** a State Key Laboratory of Pathogen and Biosecurity, Beijing Institute of Microbiology and Epidemiology, Beijing, People’s Republic of China; b Chinese PLA Center for Disease Control and Prevention, Beijing, People’s Republic of China; c Mudanjiang Forestry Central Hospital, Mudanjiang, Heilongjiang Province, People’s Republic of China; d Utrecht Centre for Tick-borne Diseases, Faculty of Veterinary Medicine, Utrecht University, Utrecht, The Netherlands; e Vector-borne Diseases Research Programme, Department of Veterinary Tropical Diseases, Faculty of Veterinary Science, University of Pretoria, Pretoria, Republic of South Africa; Yale University School of Medicine

**Keywords:** rickettsiosis, pathogenesis, skin biopsy specimens, transcriptome, *Rickettsia*

## Abstract

Spotted fever group rickettsia (SFGR) can cause mild to fatal illness. The early interaction between the host and rickettsia in skin is largely unknown, and the pathogenesis of severe rickettsiosis remains an important topic. A surveillance of SFGR infection by PCR of blood and skin biopsy specimens followed by sequencing and immunohistochemical (IHC) detection was performed on patients with a recent tick bite between 2013 and 2016. Humoral and cutaneous immunoprofiles were evaluated in different SFGR cases by serum cytokine and chemokine detection, skin IHC staining, and transcriptome sequencing (RNA-seq). A total of 111 SFGR cases were identified, including 79 “*Candidatus* Rickettsia tarasevichiae,” 22 Rickettsia raoultii, 8 Rickettsia sibirica, and 2 Rickettsia heilongjiangensis cases. The sensitivity to detect SFGR in skin biopsy specimens (9/24, 37.5%) was significantly higher than that in blood samples (105/2,671, 3.9%) (*P* < 0.05). As early as 1 day after the tick bite, rickettsiae could be detected in the skin. R. sibirica infection was more severe than “*Ca*. Rickettsia” and R. raoultii infections. Increased levels of serum interleukin-18 (IL-18), IP10, and monokine induced by gamma interferon (MIG) and decreased levels of IL-2 were observed in febrile patients infected with R. sibirica compared to those infected with “*Ca*. Rickettsia.” RNA-seq and IHC staining could not discriminate between SFGR-infected and uninfected tick bite skin lesions. However, the type I interferon (IFN) response was differently expressed between R. sibirica and R. raoultii infections at the cutaneous interface. It is concluded that skin biopsy specimens were more reliable for the detection of SFGR infection in human patients although the immunoprofile may be complicated by immunomodulators induced by the tick bite.

## Abstract (precis)

Spotted fever group rickettsia (SFGR) can cause mild to fatal illness. The early interaction between the host and rickettsia in skin is largely unknown, and the pathogenesis of severe rickettsiosis remains an important topic. A surveillance of SFGR infection by PCR of blood and skin biopsy specimens followed by sequencing and immunohistochemical (IHC) detection was performed on patients with a recent tick bite between 2013 and 2016. Humoral and cutaneous immunoprofiles were evaluated in different SFGR cases by serum cytokine and chemokine detection, skin IHC staining, and transcriptome sequencing (RNA-seq).

## INTRODUCTION

Pathogenic members of the *Rickettsia* genus are Gram-negative, obligate intracellular bacteria that have a life cycle which involves both an arthropod vector and a host (1). Spotted fever group rickettsiae (SFGR) belong to one pathogenic clade of *Rickettsia* and are mainly transmitted by ticks. The spectrum of rickettsial diseases ranges from mild influenza-like illness to life-threatening disease (2). The pathogenic events that occur in rickettsial infection begin with the entry of bacteria transmitted by the feeding tick. The initial target cells are CD68^+^ cells, including macrophages and dendritic cells (3). Then, the organism spreads throughout the body and infects mainly endothelial cells lining small and medium-sized blood vessels (4). Most of the clinical characteristics of rickettsial diseases are attributed to the disseminated infection of endothelial cells (1). Skin has long been recognized as a physical barrier providing protection from injury. As a first line of defense, skin possesses an abundant population of cells and molecular mediators of innate and adaptive immunity, resulting in what is recognized as the skin immune system (5). After a tick bite, skin manifestations of rickettsia-infected cases include skin eschar, papules, maculopapular rash, or hemorrhagic rash with petechiae (6). A skin lesion is the primary site for rickettsial introduction, and the gene expression of the bacterium itself has evolved many strategies to survive in infected skin (7). However, global gene profiling of infected skin, which reflects the host defense response to both rickettsia and tick bites, has not been extensively evaluated. Elucidating why some SFGR species are pathogenic, whereas others are not, and whether some species cause severe illness while others only cause a mild disease remain important issues. The actin-based motility mediating cell-to-cell spreading has been considered to affect rickettsia virulence (8–10). The ability to proliferate in macrophage-like cells was thought to be another explanation for why some SFGR species were nonpathogenic (11). Many more aspects of the pathogenesis of severe rickettsiosis need to be explored, in particular, the immunoprofiles of the skin lesions infected by pathogenic *Rickettsia*.

In mainland China, four major pathogenic SFGR species have been reported: R. sibirica subspecies *sibirica* BJ-90, “*Candidatus* Rickettsia tarasevichiae,” Rickettsia raoultii (12), and Rickettsia heilongjiangensis (as well as Rickettsia japonica [13], which recently was recognized as the same species as R. heilongjiangensis [14]). Mudanjiang Forestry Central Hospital is located in Heilongjiang Province in northeast China, where we identified the first five “*Ca*. Rickettsia” cases and two R. raoultii cases in 2012 (15, 16). In this study, we conducted an SFGR surveillance in the hospital between 2013 and 2016 with the aim of differentiating the humoral and cutaneous immunoprofiles of clinical SFGR cases involving different *Rickettsia* species.

## RESULTS

### Epidemiological and clinical features.

From 2013 to 2016, a total of 2,680 participants who had a history of a tick bite enrolled at the Mudanjiang Forestry Central Hospital and were screened for SFGR infection. A total of 111 patients were found to be infected with SFGR by PCR for both *gltA* and *ompA*, either from blood or eschar samples. Amplicons from all positive samples were then sequenced for confirmation. The genetic sequences were compared using BLAST (http://blast.ncbi.nlm.nih.gov/Blast.cgi), which revealed 79 “*Ca*. Rickettsia,” 22 R. raoultii, 8 R. sibirica, and 2 R. heilongjiangensis cases of infection (see Table S1 and Fig. S1 in the supplemental material). The serologic test on available samples provided supportive evidence of SFGR infection (Table S2). Seven “*Ca*. Rickettsia” patients were coinfected with other tick-borne pathogens, including “Anaplasma capra” (2 cases), Babesia crassa-like (1 case) (17), Borrelia miyamotoi (2 cases) (18), tick-borne encephalitis virus (TBEV) (1 case), and Lyme (1 case). Three R. raoultii patients were coinfected with *Babesia crassa*-like (17). R. sibirica infection was more severe than that with “*Ca*. Rickettsia” and R. raoultii. (Table S3).

### Serum cytokine and chemokine comparisons.

**FIG 1:**
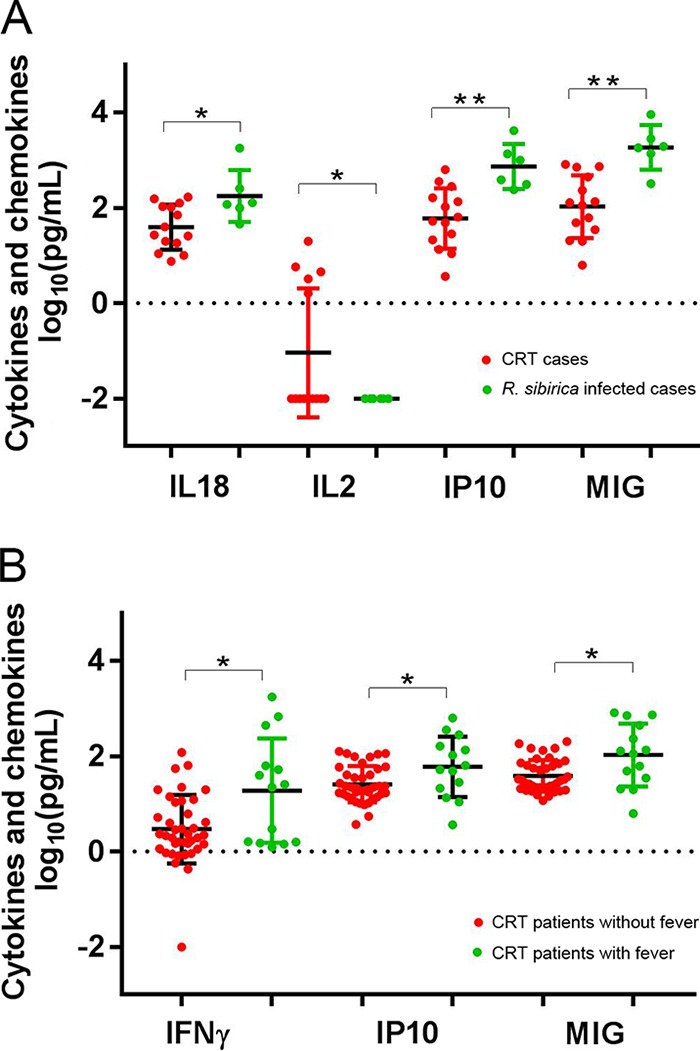
Significantly different levels of expression of serum cytokine or chemokine in “*Ca*. Rickettsia” patients with and without fever (A) and in febrile patients with “*Ca*. Rickettsia” or *R. sibirica* infection (B). *, *P* < 0.05; **, *P* < 0.01. CRT, “*Candidatus* Rickettsia tarasevichiae.”

“*Ca*. Rickettsia” infections had increased levels of fractalkine, alpha interferon (IFN-α_, IFN-γ, interleukin-10 (IL-10), IL-12, and IL-13, whereas R. sibirica infections showed elevated levels of fractalkine, IFN-γ, IL-10, IL-12, IL-18, IL-6, IP-10, monocyte chemoattractant protein 1 (MCP-1), monokine induced by gamma interferon (MIG), and tumor necrosis factor alpha (TNF-α) compared to the control group (Table S4). We then compared the cytokine and chemokine levels between patients with fever (*n* = 11) and those without (*n* = 40) within the “*Ca*. Rickettsia” infection group and found that the levels of IFN-γ, IP-10, and MIG were significantly higher in patients with fever (*P* < 0.05) (Fig. 1A). All R. raoultii patients involved in the cytokine evaluation had no fever, whereas all *R. sibirica* patients displayed fever. Increased levels of IL-18, IP10, and MIG and a decreased level of IL-2 were found in *R. sibirica* febrile patients compared with levels in “*Ca*. Rickettsia” febrile patients (Fig. 1B).

### Characterization of skin biopsy specimens.

**FIG 2:**
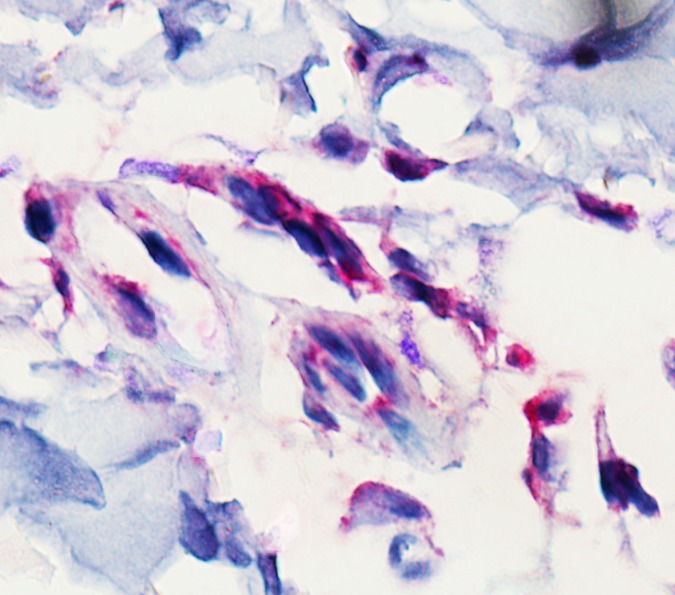
Immunohistochemical detection (hematoxylin counterstain) of Rickettsia raoultii in the skin biopsy specimen of a patient with *R. raoultii* infection. Original magnification, ×1,000.

**FIG 3:**
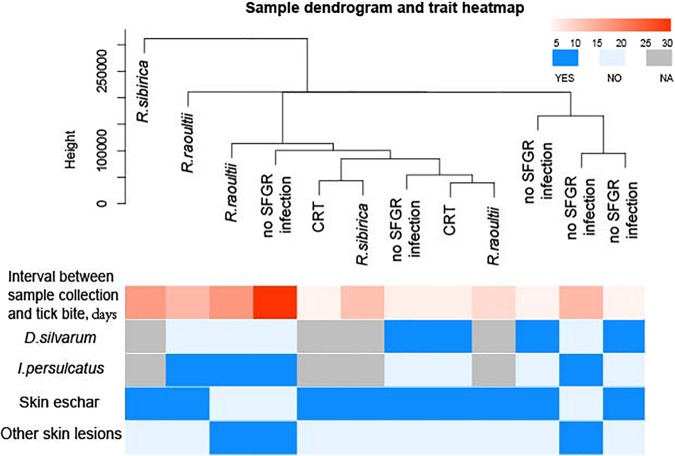
Hierarchical clustering analysis of overall gene expression by transcriptome evaluation on tick-fed skin biopsy specimens from SFGR-infected or uninfected patients. Fed-tick information, the interval between tick bite and skin sample collection, and the skin biopsy specimen type were included in the clustering analysis. NA, not available. D. silvarum, Dermacentor silvarum; I. persulcatus, Ixodes persulcatus.

A total of 24 skin biopsy specimens were collected and screened for SFGR infection. Nine positive samples were identified, including two R. sibirica, three “*Ca*. Rickettsia,” and four R. raoultii (Fig. S1). Testing of the blood samples of these nine patients revealed three positives. The sensitivity to detect SFGR in skin biopsy specimens (9/24, 37.5%) was significantly higher than that in blood samples (105/2671, 3.9%) (*P* < 0.05). As early as 1 day after the tick bite, rickettsiae could be detected in the skin samples (Table S5). The rickettsial antigen was observed in the endothelium organized in and around blood vessels (Fig. 2). Fourteen skin samples were further evaluated by transcriptome analysis, including one skin papule, four eschars, and nine tick bite skin lesions (Table S5). Seven were positive for SFGR infection (two R. sibirica, three R. raoultii, and two “*Ca*. Rickettsia”). Factors contributing to the SFGR infection, such as the interval between skin biopsy specimen collection and the tick bite, the tick species involved, and the type of skin lesion, were considered in the gene expression clustering analysis. For patients with a tick bite, dermal transcriptional activities in local lesions did not seem to be classified by the SFGR infection, based on the overall gene expression hierarchical clustering between the SFGR-infected and uninfected specimens (Fig. 3).

**FIG 4:**
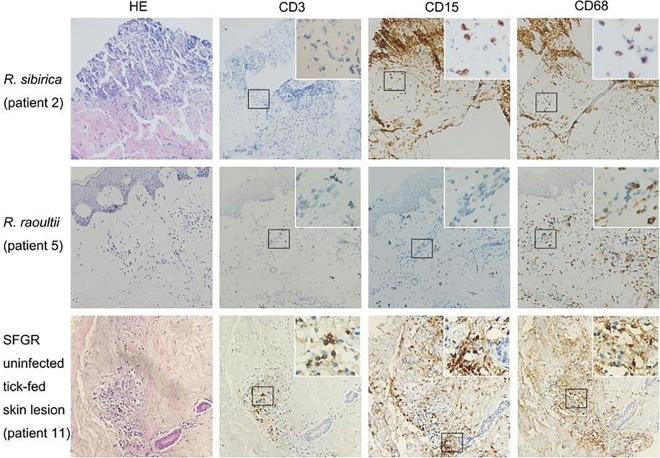
Hematoxylin and eosin (HE) staining, and immunophenotyping by lymphocyte (CD3), neutrophils (CD15), and macrophages (CD68) on serial sections of samples from patients 2, 5, and 11 (see Table S3 in the supplemental material). Magnification, ×200; inset magnification, ×400. Peroxidase (brown) was used for immunostaining.

Among the 14 patients with transcriptome sequencing (RNA-seq) data, immunohistochemical (IHC) staining was performed on rickettsia-infected (two R. sibirica, one R. raoultii, and one “*Ca*. Rickettsia” infection) and noninfected tick bite lesions (*n* = 5). No clear inflammatory infiltration was observed in the skin biopsy specimen with “*Ca*. Rickettsia” infection, collected only 1 day after the tick bite (Table S5). Skin lesions with *R. sibirica* or *R. raoultii* infection showed vascular injury and inflammatory infiltration. Macrophages infiltration occurred in *R. sibirica* and *R. raoultii* infection, but also in skin lesions without SFGR infection. Neutrophil infiltration seemed to be more prominent in *R. sibirica*-infected skin than in *R. raoultii*-infected skin but was also present in samples without SFGR infection (Fig. 4).

### Comparison of gene expression profiles between *R. sibirica*- and *R. raoultii*-infected skin biopsy specimens.

Gene ontology (GO) enrichment analysis indicated that genes associated with the type I interferon signaling pathway were significantly upregulated in R. sibirica-infected lesions compared to levels in R. raoultii-infected lesions (Table S6). All the significantly differentially regulated genes, with fold change values, are summarized in Table 1.

**TABLE 1:** Transcripts of the type I pathway induced in skin biopsy specimens of R. sibirica infection relative to levels with R. raoultii infection

Gene name	Description	Relative expression in:^*a*^											
		s1 vs r1		s1 vs r2		s1 vs r3		s2 vs r1		s2 vs r2		s2 vs r3	
		*P* value	Log_2_ FC	*P* value	Log_2_ FC	*P* value	Log_2_ FC	*P* value	Log_2_ FC	*P* value	Log_2_ FC	*P* value	Log_2_ FC
IFIT2	IFN-induced protein with tetratricopeptide repeats 2	2.40E−09	8.78787	1.41E−08	3.36166	9.67E−07	2.25908	2.20E−64	16.87193	1.84E−70	12.28032	5.72E−37	6.703528
RSAD2	Radical *S*-adenosyl methionine domain containing 2	4.78E−07	8.204539	9.92E−11	5.268731	1.38E−08	2.71806	5.45E−62	16.55291	1.42E−71	14.43458	1.57E−35	6.509623
ISG15	ISG15 ubiquitin-like modifier	0.002435	6.457162	0.000194	4.411335	2.68E−07	2.88902	5.20E−45	14.27856	1.71E−54	13.03666	6.48E−30	5.816973
IFIT3	IFN-induced protein with tetratricopeptide repeats 3	9.35E−18	10.37792	1.07E−13	3.903858	0.032548	0.92007	6.54E−59	16.14215	3.11E−60	10.50377	7.95E−30	5.721682
MX2	MX dynamin-like GTPase 2	3.76E−05	2.118935	2.57E−12	3.735954	0.000906	1.44553	6.00E−42	7.883568	10.18116	1.47E−57	5.49E−25	5.041784
IFIT1	IFN-induced protein with tetratricopeptide repeats 1	8.49E−10	8.872573	2.89E−14	5.937531	1.40E−07	2.42459	5.47E−51	15.0825	1.59E−60	12.96415	2.66E−22	4.663792
OASL	2'-5'-Oligoadenylate synthetase like	7.40E−07	7.875647	2.01E−07	3.994897	0.018371	1.16953	3.06E−33	12.66724	1.82E−42	9.614755	1.90E−20	4.501635
GBP2	Guanylate binding protein 2 IFN inducible	4.09E−21	4.897447	0.000739	1.427524	0.004756	1.185573	2.81E−34	6.762797	1.19E−17	3.97486	2.32E−16	3.772888
HLA-C	Major histocompatibility complex class I, C	6.79E−22	6.31561	0.01114	1.08249	0.010307	1.07963	6.00E−44	9.434273	1.10E−23	4.886665	3.00E−10	2.764606
OAS3	2'-5'-Oligoadenylate synthetase 3	2.67E−21	6.227064	0.003659	1.246947	0.001064	1.38771	3.63E−42	9.193872	1.02E−23	4.899251	9.12E−08	2.304688
IFITM1	IFN-induced transmembrane protein 1	1.08E−12	9.483692	0.010353	1.200392	0.025493	0.9894	2.44E−24	11.39066	6.93E−16	3.944462	3.68E−05	1.795146
OAS2	2'-5'-Oligoadenylate synthetase 2	4.41E−18	6.482564	2.33E−14	3.91359	0.000492	1.49385	1.22E−33	8.769977	1.48E−34	6.889158	0.000318	1.523028
PTPN1	Protein tyrosine phosphatase nonreceptor type 1	0.000784	1.659851	0.013215	1.118948	0.039233	0.89303	2.67E−09	2.941685	3.16E−11	3.08255	0.009438	1.110821

a The comparisons are indicated as follows: s1 and s2 represent R. sibirica-infected cases 1 and 2, respectively; r1, r2, and r3 represent R. raoultii-infected cases 1, 2, and 3, respectively. FC, fold change in expression level.

## DISCUSSION

SFG rickettsiosis is a worldwide tick-borne infectious disease in humans, with clinical symptoms varying from mild to fatal illness. SFGR infection is difficult to diagnose since the early signs and symptoms are nonspecific, and acute-phase diagnostic tests are not widely available (19). Moreover, a single IgG antibody titer is an unreliable measure for the diagnosis of SFGR infection (20). Through surveillance in our sentinel hospital from 2013 to 2016, we were able to confirm a total of 111 (4%, 111/2680) SFGR cases by SFGR-specific gene PCR and sequencing on acute blood and skin eschar samples. The skin is the first human organ that tick saliva and tick-borne pathogen encounter during the tick feeding process. However, the early response of rickettsiae at the cutaneous interface is largely unknown. We found that the sensitivity to detect SFGR in skin biopsy specimens was significantly higher than in blood samples, indicating that many more cases of SFGR infection may be missed due to limitations in the diagnostic method. In animal models, the testing of skin samples has also resulted in a higher proportion of positive results than the testing of blood samples (21). Molecular detection of SFG rickettsiae in noninvasive skin biopsy specimens could therefore become a major innovation in the diagnosis of SFG rickettsioses (22, 23). Rickettsiae could be detected as early as 24 h after a tick bite, which is sooner than detection of the causal pathogen of Lyme disease, Borrelia burgdorferi *sensu stricto* (24). R. raoultii has been associated with a syndrome characterized by scalp eschar and neck lymphadenopathy following tick bites (SENLAT) (25). Here, we demonstrated detection of R. raoultii in the endothelial cells of blood vessels in skin lesions using an immunohistochemical method.

We used molecular diagnostic tests to identify the causative agents and found four SFGR species causing illness in northeastern China. R. sibirica infection appeared to be most severe, with the highest frequency of fever and general rash. The pathogenesis which differentiates mild and severe rickettsioses is incompletely understood although the immune system is known to play a key role (26). The observed increase in cytokines, including monokine induced by IFN-γ (MIG), IP10 (IFN-γ-inducible 10-kDa protein), and IL-18 (also known as IFN-γ-inducing factor), indicate that an IFN-γ-related mechanism is contributing to the pathogenesis of severe rickettsioses. IFN-γ could activate human endothelial cells and macrophages, eliciting the intracellular killing of rickettsiae (27). It is worth mentioning that the severity of disease has been reported to correlate with survival and proliferation of rickettsiae in human macrophage-like cells, which mediate the clearance of pathogens in response to IFN-γ (11). Furthermore, IFN-γ is also the major effector of the adaptive immune response to rickettsial infection; IFN-γ knockout mice are more than 100-fold more susceptible than wild-type mice to infection with Rickettsia australis (28). On the other hand, we observed the downregulated level of serum IL-2 in R. sibirica-infected cases. An immune-suppressed CD4 T cell response has been reported as a component of the pathogenesis of severe rickettsioses, which is linked to the failure to produce IL-2 (29).

Ixodid ticks are unique in that they attach to the host skin and blood feed for several days, allowing a diverse range of biologically active molecules expressed in tick saliva to modulates the host’s cutaneous defense mechanism (30). The knowledge of changes at the cutaneous interface between the tick, rickettsiae, and the host is limited. The cellular infiltrate reported in biopsy specimens of eschars from human cases of R. conorii (31), R. japonica (32), and R. parkeri (33) rickettsiosis predominantly consists of macrophages and lymphocytes, whereas neutrophils predominate in eschars of R. africae-infected patients (34). We observed macrophage infiltration in both R. sibirica- and R. raoultii-infected skin lesion biopsy specimens, whereas neutrophils were more related to the cutaneous response in *R. sibirica* infections. The cellular difference in infiltration might be related to the different *Rickettsia* species. However, the saliva from different tick species could also alter the cutaneous cellular immune response. For instance, neutrophils are recognized as the most abundant cells in the acute inflammatory infiltrate induced by the tick primary infestation (35). Indeed, we also observed neutrophil recruitment in tick bite lesions without rickettsia infection. The immunoprofile is therefore complicated by immunomodulators induced by the tick bite. The cutaneous cellular influx during the acute phase of R. parkeri infection in C3H/HeN mice could be altered after coinoculation with Amblyomma maculatum saliva (36). Compared to levels in normal skin samples, the intralesional mRNA expression levels of TNF-α, IFN-γ, RANTES, indoleamine 2,3-dioxygenase (IDO), and inducible nitric oxide synthase (iNOS) were significantly upregulated in Mediterranean spotted fever rickettsia patients (37). However, the tick bite and its related factors could influence the cutaneous transcriptome level since the intralesional gene expression profiles in this study could not be clustered and classified based on the single characteristic of SFGR infection. The histochemical and transcriptomic manifestations in the cutaneous interface of SFGR patients may be influenced by the tick as well as by the intradermal inoculation of rickettsiae.

The pathogenesis of severe rickettsiosis remains an important topic for SFGR infection prevention and control. Through the transcriptome assessment of patients’ skin lesions infected with R. sibirica and R. raoultii, we found that *R. sibirica*, which caused more severe illness, induced higher type I interferon pathway responses at the cutaneous interface. The type I interferon response has been recognized as a first-line defense mechanism for tick-borne flaviviruses (38). Recently, a study revealed that human microvascular endothelial cells (HMECs) infected with R. conorii also launch “antiviral” host defense mechanisms typically governed by type I interferons and display increased expression of IFN-stimulated genes (ISGs), for example ISG15, which activate innate responses to interfere with the intracellular replication of rickettsiae (39–41). By differential expression analysis, we found that the ISG15 transcript level was upregulated in R. sibirica-infected skin biopsy specimens. Petzke et al. reported that a Borrelia burgdorferi strain causing dissemination in a murine model induced a higher type I interferon response in skin lesions than a *Borrelia* strain without the capacity for dissemination (42). Whether the upregulated type I interferon response stimulated by R. sibirica in local skin will be correlated with its disseminated infection and cause further severe symptoms deserves further evaluation.

This work has the following limitations. First, the sample size for skin biopsy specimens of each *Rickettsia* species is relatively small. Second, the collection methodologies used for the skin biopsy specimens may have affected the transcriptome profile; thus, more samples are required to stratify and limit confounding factors. However, to our knowledge, this study is the first to use global expression profiling to characterize the human skin transcriptome during early *Rickettsia* infection.

In conclusion, immunoprofiling of the early events in human skin after the bite of rickettsia-infected ticks contributes to our understanding of the early pathogenesis of spotted fever group rickettsiosis and has potential for improved diagnostics.

## MATERIALS AND METHODS

### Patient enrollment.

We conducted a continuous SFGR infection surveillance in Mudanjiang Forestry Central Hospital from April to June during 2013 to 2016. Patients with a history of a tick bite were enrolled, and a standard questionnaire and medical records containing the necessary information were recorded as described previously (43). All participants provided written informed consent in this study, which was approved by the Mudanjiang Forestry Central Hospital Review Board and Academy of Military Medical Sciences Review Board, China.

### Sample collection and DNA/RNA extraction.

EDTA-anticoagulated blood and serum samples were collected on the first day that the patients consulted a physician. Convalescent serum samples were requested at least 14 days and at most 2 months after the onset of acute illness. During the surveillance season in 2016, skin biopsy specimens were collected from enrolled patients. Punch biopsy specimens (TianNuoTianCheng, Inc., Beijing, China) from skin papules and from the eschar were taken after local anesthesia with 1% lidocaine. Some patients with a tick bite consulted their physicians. In these cases, the tick was carefully removed by forceps, and a skin biopsy specimen was taken near the site of the tick bite. The biopsy specimens were cut into two pieces using a sharp scalpel; one was transferred into an RNase-free tube containing Sample Protector for RNA/DNA (TaKaRa, Dalian, China) and stored in liquid nitrogen, and the second half of each skin biopsy specimen was used for bacterial culture, PCR, and immunohistochemical staining after fixation in 10% formalin.

### DNA and RNA extraction.

DNA was extracted from whole blood (QIAmp DNA Blood Mini kit; Qiagen, Shanghai, China) according to the manufacturer’s instructions. Total DNA and RNA extraction from skin biopsy specimens was performed using an AllPrep DNA/RNA Mini kit (Qiagen) with some modifications. Briefly, skin tissue samples were decontaminated with iodated alcohol after being recovered from the RNA/DNA sample protector. The tissues were briefly washed in RNase-free water twice and then homogenized in RLT solution (Qiagen) under liquid nitrogen. The homogenate was then incubated at 55°C for 10 min with proteinase K (Qiagen) and centrifuged for 30 s at full speed. The homogenized lysate was transferred to an AllPrep DNA spin column and centrifuged for 30 s at 12,000 × *g*. An AllPrep DNA spin column was used for later DNA purification, and the flowthrough was used for RNA purification, according to the manufacturer’s instructions.

### PCR and indirect IFA.

All DNA was screened by PCR specific for the conserved citrate synthase gene (*gltA*), and positive samples were further confirmed by PCR for the spotted fever group-restricted outer membrane protein A gene (*ompA*). All positive amplicons were sequenced to identify the SFGR species as described in previous reports (15, 16, 44). Serum samples were tested by immunofluorescence assay (IFA) for IgG antibodies against R. heilongjiangensis, which cross-reacts with R. raoultii, “*Ca*. Rickettsia,” and R. sibirica (15, 16, 44). Coinfection with other tick-borne pathogens was identified as described previously (43).

### Cytokine and chemokine detection.

Serum cytokines and chemokines, fractalkine (CX3CL1), gamma interferon (IFN-γ), IFN-α, interleukin-12 p70 (IL-12p70), IL-13, IL-1b, IL-6, IL-8, IL-18, IL-2, IL-10, tumor necrosis factor α (TNF-α), monocyte chemoattractant protein 1 (MCP-1), monokine induced by IFN-γ (MIG), macrophage inflammatory protein 1α (MIP-1α), MIP-1β, IFN-γ-inducible 10-kDa protein (IP10), and the stromal cell-derived factor 1α (SDF-1α) were analyzed using a multiplex Luminex assay (ProcartaPlex kit; ThermoFisher, Waltham, MA, USA) according to the manufacturer’s recommendations. Serum samples from healthy residents (*n* = 51) of similar ages and sex from the same region were tested simultaneously. All samples were stored at –40°C and tested in batches in May 2017.

### Immunohistochemical staining.

Hematoxylin-eosin staining was performed according to standard methods (45) on formalin-fixed, paraffin-embedded skin biopsy specimens. Serial sections (5-μm thickness) were also used for immunohistochemical (IHC) staining. IHC examination for SFGR was performed according to the method described by Dumler et al. (45). Briefly, deparaffinized slides were incubated with mouse polyclonal antibody against R. heilongjiangensis (1:100) for 1 h at room temperature (46). *R. heilongjiangensis* was isolated from ticks in Heilongjiang Province where the study was carried out (47) and was cross-reacted with R. raoultii, “*Ca*. Rickettsia,” and R. sibirica (15, 16, 44). After a washing step, each section was incubated with biotinylated goat anti-mouse IgG (Abcam, Cambridge, MA, USA) for 30 min at room temperature. Then, sections were incubated with alkaline phosphatase-conjugated streptavidin (R&D Systems, Minneapolis, MN, USA), followed by Fast-Red substrate (R&D Systems). The slides were counterstained with Mayer’s hematoxylin (ZSGB-Bio, Beijing, China) for 10 min. To characterize the immune response in skin lesions, deparaffinized sections were stained with the T-lymphocyte marker CD3 (Abcam), the polymorphonuclear leukocyte marker CD15 (Abcam), and the macrophage marker CD68 (Abcam) using a peroxidase-based method developed in our own laboratories (48).

### Transcriptome sequencing.

Total RNA was used for transcriptome sequencing after RNA quantification and qualification (Qubit RNA assay kit) in a Qubit 2.0 fluorometer (Life Technologies, Waltham, MA, USA). A total of 500 pg of qualified RNA per sample was used as input material for the RNA sample preparations. Sequencing libraries were generated using an Ovation SoLo RNA-Seq System, Human (NuGEN, Redwood City, CA, USA), according to the manufacturer’s recommendations. Paired-end (150 bp) sequencing of the RNA library was performed on an Illumina HiSeq 4000 platform.

### Hierarchical clustering analysis from sample gene expression.

The gene expression levels were first normalized using DEseq (49). The normalized expression levels from all genes were used to conduct hierarchical clustering across all of the samples. The hierarchical clustering tree and sample trait annotation matrix were plotted using the WGCNA package (50).

### Differential expression analysis and GO enrichment.

Differential expression analysis between each individual with R. sibirica infection and each individual with R. raoultii infection was performed using edgeR (51), resulting in six pairwise comparisons. Then, gene ontology (GO) enrichment analysis was conducted based on the significantly differentially expressed (DE) genes in each comparison. The enriched GO terms were aggregated across six comparisons, and a GO term was considered significant when six *P* values were all less than 0.05. The DE genes were identified when the *P* values were all less than 0.05 when individual R. sibirica cases were compared to individual R. raoultii ones.

### Statistical analysis.

Continuous variables were summarized as median and range, and categorical variables were summarized as frequencies and proportions. The values of serum levels of cytokines and chemokines were log transformed. For variables that were not normally distributed, comparisons were made using a Mann-Whitney U test. A two-sided *P* value of less than 0.05 was regarded as significant. All the analyses were performed using SPSS software (version 18.0).

### Data availability.

All of the transcriptome data obtained in this study have been submitted to the Gene Expression Omnibus data repository under accession number GSE141235.

## Supplementary Material

Supplemental file 1

Supplemental file 2
