# Supplementary material for: Cutaneous Immunoprofiles of Three Spotted Fever Group Rickettsia Cases
Source: Infect Immun. 2020 Mar 23;88(4):e00686-19. doi: 10.1128/IAI.00686-19 (PMC7093127; doi:10.1128/IAI.00686-19)
Supplement: Supplemental file 1 [file IAI.00686-19-s0001.pdf]

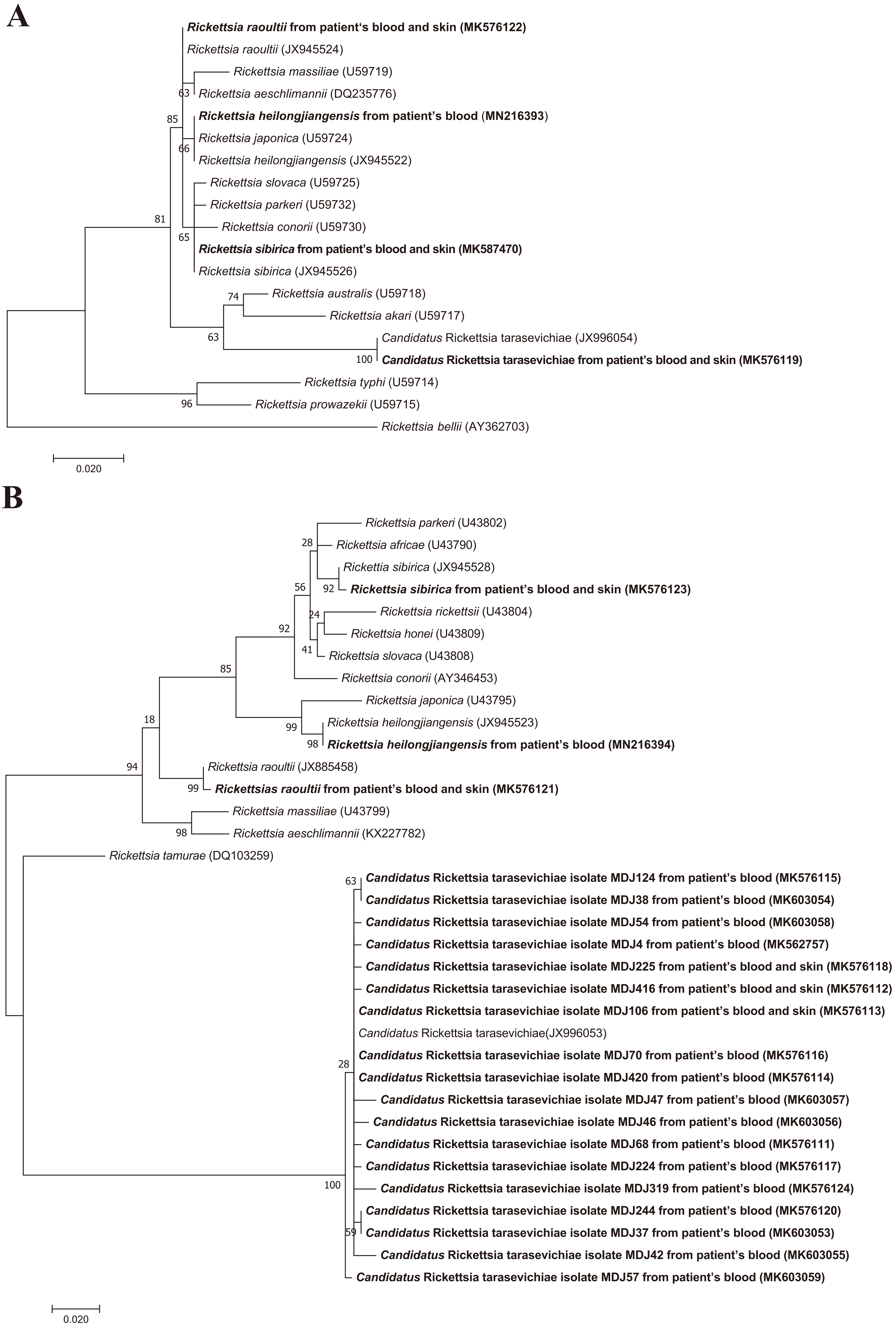

The nucleotide sequences determined in this study are indicated in bold in each phylogenetic tree. Panel A shows the phylogenetic tree based on partial (305 bp) citrate synthase coding gene (gltA), obtained by using the neighbor-joining method and with distances calculated by means of the Kimura 2-parameter analysis as the number of base substitution per site. The percentage of replicate trees in which the associated taxa clustered together in the bootstrap test (of 1000 replicates) is shown to the left of each branch. Panel B shows phylogenetic tree based on partial (328 bp) outer membrane protein A coding gene (ompA) with the same method as mentioned above.
