# Supplementary material for: Cutaneous Immunoprofiles of Three Spotted Fever Group Rickettsia Cases
Source: Infect Immun. 2020 Mar 23;88(4):e00686-19. doi: 10.1128/IAI.00686-19 (PMC7093127; doi:10.1128/IAI.00686-19)
Supplement: Supplemental file 2 [file IAI.00686-19-s0002.pdf]

Supplemental Table S1. Genetic similarity of Spotted Fever Group Rickettsiae sequences obtained from patients with validated *Rickettsia spp*. Infections, in Mudanjiang, China. Data published in GenBank.

Genes	Sequence length (bp)	Sequence identity (GenBank number)			
		CRT	*R. raoultii*	*R. sibirica*	*R. heilongjiangensis*
gltA	382	100 % to CRT (JX996054)	100 % to *R. raoultii* (JX945524)	100 % to *R. sibirica* (JX945526)	100 % to *R. heilongjiangensis* (JX945522)
ompA	348	99.4 %–100 % to CRT (JX996053)	99.4 % to *R. raoultii* (JX885458)	99.4 % to *R .sibirica* (JX945528)	100 % to *R. heilongjiangensis* (JX945523)

CRT, *Candidatus* Rickettsia tarasevichiae

Supplemental Table S2. Antibody Titres Against *Rickettsia heilongjiangensis* in serum samples from the patients*

Patient	SFGR species infecting patient	Time of sample collection#, day	IFA Titre for *R. heilongjiangensis***	Time of sample collection, day	IFA Titre for *R. heilongjiangensis*
1	CRT	3	<64	15	1024
2	CRT	4	<64	41	64
3	CRT	1	<64	29	64
4	CRT	6	<64	36	128
5	CRT	7	<64	36	64
6	CRT	3	64	33	256
7	CRT	1	256	41	512
8	CRT	5	64	22	256
9	CRT	3	<64	40	128
10	CRT	5	128	23	512
11	CRT	5	<64	35	64
12	*R. heilongjiangensis*	5	64	15	1024
13	*R. heilongjiangensis*	3	64	13	2048
14	CRT	4	<64	26	128
15	CRT	4	<64	26	64
16	CRT	5	64	18	512
17	CRT	14	<64	39	64
18	CRT	3	<64	26	128
19	CRT	7	<64	27	64
20	CRT	1	64	23	256
21	CRT	2	64	21	128
22	CRT	1	<64	20	64

23	*R. raoultii*	7	<64	133	64
24	*R. sibirica*	2	<64	125	512
25	*R. sibirica*	17	<64	106	512
26	*R. raoultii*	3	<64	17	512
27	*R. raoultii*	6	64	20	512
28	*R. raoultii*	5	<64	35	64
29	*R. raoultii*	2	<64	30	64

*29 patients provided serial serum samples for serological testing

#Number of days after the onset of illness for patients

**IFA, immunofluorescence assay, titres are the reciprocals of the serum dilution

CRT, *Candidatus* Rickettsia tarasevichiae

Supplemental Table S3. Epidemiological, Clinical Characteristics, and Laboratory Test Results of Patients with SFGR Infections

Characteristics		Cases, n (%)		
		CRT (n=79)	*R. raoultii* (n=22)	*R. sibirica* (n=8)
**Epidemiological characteristics**				
Sex, female (%)		47 (59)	10 (45)	2 (25)
Age (range), year		49 (3–79)	52 (28–77)	53 （27–67）
Tick bite location				
	Scalp, ears, or neck	32 （40）	9 （41）	2 （25）
	Trunk	21 (27)	7 （32）	4 （50）
	Extremities	15 (19)	4 （18）	1 （13）
	Multiple	11 (14)	2 （9）	1 (13)
Place of exposure to ticks				
	Forest	36 (46)	7 （32）	1 （13）
	Courtyard	18 (23)	4 （18）	1 （13）
	Farm	13 (16)	0	2 （25）

	Others	5 (6)	0	1 （13）
	Unknown	7 (9)	11 （50）	3 （38）
Month of symptom onset	April	5 (6)	4 （18）	2 （25）
	May	68 (86)	17 （77）	6 （75）
	June	6 (8)	1 （5）	0
**Clinical manifestation**				
Fever		17 （22）	6 （27）	7 （86）
Highest temperature (°C) mean±SD		38.5±0.61	38.9±0.88	39.3±0.65
Asthenia		13 (16)	1 (5)	4 （50）
Myalgia		3 (4）	3 (14)	5 （62）
Gastrointestinal symptoms		8 (10）	2 (9)	1 (13)
Headache		28 (35）	6 (27)	5 (62）
Skin lesions				
	Papules/ maculopapular/macules rash	9 （11）	5 (23)	6 （75）

Eschar	11 （14）	4 (18）	0 （0）
Lymphadenopathy	9 （11）	3 (14）	1 （13）
Hospitalisation	8 （10）	5 (23)	4 （50）
Days between known tick bite and any symptom or sign onset, median (range)	3 (0–23)	2 (0–8)	7 (0–12)
**Haematologic test**			
Leukocyte count<4 $\times 10^9$/L	8 /67(12)	4 /16(25)	1/6 (20)
Leukocyte count>10 $\times 10^9$/L	6 /67(9)	1/16 (6)	2 /6(25)
Thrombocytopenia	6 /67(9)	0	1/6 (20)
Anaemia	6 /67(9)	0	0
Lymphocytopenia	5/67 (7)	2/16 (13)	2 /6(40)
Elevated neutrophil count	6/67 (9)	0	2 /6(40)
**Chemical test**			
Aspartate aminotransferase>40 U/L	5 /50(10)	2 /15(13)	5/6 (62)
Alanine aminotransferase>40 U/L	8 /50(16)	3 /15(20)	5 /6(62)

CRT, *Candidatus* R. tarasevichiae

Supplemental Table S4. Serum Cytokine and Chemokine Comparisons of Three SFGR Species

Cytokine and Chemokines (log_{10} pg/ml)	Control group (n=51)	Rickettsia group			CRT vs Control	*R .raoultii* vs Control	*R. sibirica* vs Control
		CRT (n=54)	*R. raoultii* (n=10)	*R. sibirica* (n=6)			
Fractalkine	0.91 (0.73–1.12)	1.15 (0.99–1.43)	0.92 (0.78–1.26)	1.30 (1.14–1.90)	**<0.0001**	0.7	**0.01**
IFNalpha	-2.00 (-2.00–-2.00)	-2.00 (-2.00–0.18)	-1.19 (-2.00–-0.22)	-1.17 (-2.00–0.03)	**0.02**	0.1	0.1
IFNgamma	0.09 （-0.21–0.35）	0.37 (0.17–1.30)	0.25 (-0.08–0.98)	2.01 (1.74–2.76)	**<0.0001**	0.3	**<0.001**
IL1 beta	-0.03 (-0.34–0.42)	0.03 (-0.18–0.71)	0.08 (-0.81–0.19)	0.21 (0.10–0.80)	0.4	0.5	0.2
IL 10	-0.27 (-1.15–0.14)	0.01 (-0.42–0.65)	-0.30 (-0.45–-0.05)	0.81 (0.21–1.48)	**0.02**	0.9	**0.03**
IL 12	-2.00 (-2.00–0.02)	0.01 (-2.00–0.57)	0.39 (-2.00–0.47)	0.82 (0.65–0.95)	**0.001**	0.07	**<0.001**
IL 13	-2.00 (-2.00–-0.02)	-0.08 (-2.00–0.57)	-2.00 (-2.00–-1.41)	-1.00 (-2.00–0.58)	**0.007**	0.3	0.5
IL 18	1.46 (1.16–1.73)	1.60 (1.30–1.93)	1.18 (-2.00–1.65)	2.09 (1.92–2.61)	0.1	0.1	**0.002**
IL 2	-2.00 (-2.00–-0.30)	-2.00 (-2.00–0.60)	-2.00 (-2.00–1.10)	-2.00 (-2.00–-2.00)	0.1	0.6	0.2
IL 6	0.54 (-2.00–1.83)	1.24 (0.12–1.77)	-2.00 (-2.00–1.43)	2.16 (1.07–2.24)	0.2	0.2	**0.03**
IL 8	1.52 (0.50–2.32)	1.82 (1.09–2.22)	1.05 (0.82–1.63)	1.66 (1.02–2.18)	0.2	0.5	0.4
IP 10	1.33 (1.07–1.49)	1.37 (1.18–1.84)	1.37 (1.11–1.69)	2.80 (2.46–3.25)	0.07	0.6	**<0.001**
MCP1	2.08 (1.87–2.28)	2.16 (2.01–2.57)	2.02 (1.82–2.29)	2.49 (2.21–2.98)	0.06	0.7	**0.01**
MIG	1.57 (1.38–1.75)	1.54 (1.34–2.01)	1.59 (1.35–1.62)	3.28 (2.98–3.58)	0.5	0.8	**0.001**
MIP1alpha	1.30 (0.82–2.11)	1.75 (1.10–2.10)	0.99 (0.87–1.60)	1.29 (0.89–2.06)	0.3	0.3	0.9
MIP1beta	2.42 (2.06–2.80)	2.50 (2.15–2.84)	2.09 (1.90–2.37)	2.53 (1.99–2.90)	0.3	0.09	0.6
SDF1alpha	2.36 (2.20–2.51)	2.46 (2.33–2.61)	2.34 (2.07–2.58)	2.45 (2.31–2.82)	0.8	0.7	0.2
TNFalpha	0.45 (0.29–1.03)	0.70 (0.28–1.16)	0.42 (-2.00–0.58)	0.96 (0.85–1.58)	0.2	0.2	**0.03**

CRT, *Candidatus* R. tarasevichiae. Data are given as log-transformed median (interquartile range)

Supplemental Table S5. Information for 24 SFGR patients who provided skin biopsy samples

No.	Skin biopsy	SFGR species	IHC evaluation	RNA-seq	Interval between sample collection and tick bite, days	Detached tick species
1	Skin papules	*R. sibirica*	Yes	Yes	9	NA
2	Tick bite skin lesion	*R. sibirica*	Yes	Yes	14	NA
3	Eschar	*R. raoultii*	NA	Yes	15	*D. silvarum*
4	Tick bite skin lesion	*R. raoultii*	NA	Yes	10	*D. silvarum*
5	Tick bite skin lesion	*R. raoultii*	Yes	Yes	5	NA
6	Tick bite skin lesion	CRT	Yes	Yes	1	NA
7	Tick bite skin lesion	CRT	NA	Yes	2	*I. persulcatus*
8	Tick bite skin lesion	Neg	NA	Yes	1	*I. persulcatus*
9	Eschar	Neg	Yes	Yes	10	NA
10	Eschar	Neg	Yes	Yes	10	*D. silvarum*
11	Tick bite skin lesion	Neg	Yes	Yes	6	*I. persulcatus*
12	Tick bite skin lesion	Neg	NA	Yes	1	*H. concinna*
13	Eschar	Neg	Yes	Yes	30	*D. silvarum*
14	Tick bite skin lesion	Neg	Yes	Yes	1	*I. persulcatus*
15	Tick bite skin lesion	Neg	NA	No	1	NA
16	Eschar	Neg	Yes	No	2	NA
17	Tick bite skin lesion	Neg	Yes	No	3	NA
18	Tick bite skin lesion	Neg	NA	No	1	NA
19	Tick bite skin lesion	CRT	NA	No	2	NA
20	Tick bite skin lesion	Neg	NA	No	3	NA
21	Tick bite skin lesion	*R. raoultii*	NA	No	1	*I. persulcatus*
22	Tick bite skin lesion	Neg	NA	No	4	NA

23	Tick bite skin lesion	Neg	Yes	No	31	NA
24	Tick bite skin lesion	Neg	NA	No	1	NA

IHC, immunohistochemial examination
NA, not available

Supplemental Table S6. Gene Ontology (GO) Enrichment Analysis of the Differential Gene Expression between *R. sibirica* and *R. raoultii* Induced Cutaneous Immune Responses

GO Descriptions	*P* value (Increased/decreased change)					
	s1 vs r1	s1 vs r2	s1 vs r3	s2 vs r1	s2 vs r2	s2 vs r3
Type I interferon signalling pathway	1.90E-07 (+)	1.10E-05 (+)	9.10E-04 (+)	6.90E-04 (+)	2.90E-04 (+)	6.00E-03 (+)
Cellular response to type I interferon	1.90E-07 (+)	1.10E-05 (+)	9.10E-04 (+)	6.90E-04 (+)	2.90E-04 (+)	6.00E-03 (+)
Defence response to virus	4.30E-04 (+)	2.90E-07 (+)	1.10E-03 (+)	2.00E-01 (+)	1.60E-04 (+)	3.60E-04 (+)
Response to virus	1.00E-04 (+)	1.00E-08 (+)	4.70E-06 (+)	5.90E-02 (+)	2.00E-04 (+)	4.50E-03 (+)
Response to type I interferon	4.20E-08 (+)	1.70E-06 (+)	1.90E-03 (+)	1.10E-03 (+)	2.90E-04 (+)	2.10E-03 (+)

s1 and s2 represent *R. sibirica* infected case 1 and case 2, respectively; r1, r2, and r3 represent *R. raoultii* infected case 1, case 2, and case 3, respectively. (+) means the changes represented increased change.
